# Outcome of girls with central precocious puberty (CPP)

**DOI:** 10.1186/1687-9856-2015-S1-P90

**Published:** 2015-04-28

**Authors:** Ruchi Shah, Sudha Rao, Meena Desai, Rajesh Joshi, Aparna Limaye, Ruchi Parikh, Poonam Singh, Neha Dighe

**Affiliations:** 1 Division of Pediatric Endocrinology, Bai Jerbai Wadia Hospital for Children, Mumbai, Maharashtra, India

## 

Due to non-affordability of GnRH analogues (GnRHa), Medroxy Progesterone Acetate (MPA) is still used as a treatment option in girls with CPP in India. The aim here is to study the clinical features of girls with CPP in respect to the age at presentation, etiology and outcome as per the medication used for their treatment (GnRHa vs MPA).

Retrospective study of 38 girls with CPP(19 idiopathic) treated with either MPA or GnRHa (Luprorelin or Triptorelin) were followed up for period varying from 1 to 8 years. The progression of growth parameters in relation to their age, etiology and medication used for the treatment was studied. Statistical analysis was done using one way ANOVA, unpaired t-test and Pearson correlation tests.

Mean age at onset of puberty in girls with idiopathic ICPP(n=19), neurogenic NCPP(n=12) and hypothalamic hamartoma(HH)(n=7) were 4.68±2.83, 5.32±2.20 and 1.76±1.44 years, respectively(P 0.003). Mean height sds at presentation of all girls was higher (0.51±1.80) with comparison to MPH sds (0.90±1.19)(P=0.0021). 70% of the girls presented with SMR stage 3. Bone age was more advanced as compared to height age in all (P=0.0001) and this trend continued till last follow up. At presentation, girls with ICPP were significantly heavier and taller as compared to NCPP and HH (P=0.002 for weight SDS and 0.022 for height SDS). Mean baseline LH, FSH and estradiol were 3.05±2.49 mIU/ml, 4.75±2.29 mIU/ml and 29.43 pg/ml; respectively. At presentation, bone age was more advanced in HH(BA/CA P=0.0001 with BA/HA P=0.03) and LH levels significantly higher(5.6±2.26; P=0.02). At last follow up, there was no significant difference in weight sds(p=0.285), height sds(p=0.074) and PAH sds(p=0.056) in girls of different age groups and etiologies. After 4 years of follow up, height sds was better with GnRHa(1.80±0.98) as compared to MPA(P=0.01) but height sds with MPA was also good(0.49±0.81).

Due to prevalence of CNS infections, the proportion of NCPP(31.57%) is higher in our set up, as compared to industrialized countries. With financial constraints, MPA can be considered as a treatment option for CPP.

